# A Novel Strategy for Enhanced Sequestration of Protein-Bound Uremic Toxins Using Smart Hybrid Membranes

**DOI:** 10.3390/jfb14030138

**Published:** 2023-02-28

**Authors:** Madalena Lopes, Rita F. Pires, Mónica Faria, Vasco D. B. Bonifácio

**Affiliations:** 1 Center of Physics and Engineering of Advanced Materials CeFEMA, Instituto Superior Técnico, Universidade de Lisboa, 1049-001 Lisboa, Portugal; 2 Institute of Bioengineering and Biosciences (iBB) and Institute for Health and Bioeconomy (i4HB), Instituto Superior Técnico, Universidade de Lisboa, 1049-001 Lisboa, Portugal; 3 Bioengineering Department, Instituto Superior Técnico, Universidade de Lisboa, 1049-001 Lisboa, Portugal

**Keywords:** monophasic hybrid membranes, sol-gel, competitive binding, protein-bound uremic toxins, chronic kidney disease, hemodialysis

## Abstract

Currently available hemodialysis (HD) membranes are unable to safely remove protein-bound uremic toxins (PBUTs), especially those bonded to human serum albumin (HSA). To overcome this issue, the prior administration of high doses of HSA competitive binders, such as ibuprofen (IBF), has been proposed as a complementary clinical protocol to increase HD efficiency. In this work, we designed and prepared novel hybrid membranes conjugated with IBF, thus avoiding its administration to end-stage renal disease (ESRD) patients. Two novel silicon precursors containing IBF were synthesized and, by the combination of a sol-gel reaction and the phase inversion technique, four monophasic hybrid integral asymmetric cellulose acetate/silica/IBF membranes in which silicon precursors are covalently bonded to the cellulose acetate polymer were produced. To prove IBF incorporation, methyl red dye was used as a model, thus allowing simple visual color control of the membrane fabrication and stability. These smart membranes may display a competitive behavior towards HSA, allowing the local displacement of PBUTs in future hemodialyzers.

## 1. Introduction

The kidney is a vital organ that removes metabolic wastes from the blood. Chronic kidney disease (CKD) leads to a gradual and irreversible long-term condition, known as end-stage renal disease (ESRD), characterized by permanently impaired kidneys which cannot filter blood efficiently. Therefore, in ESRD, harmful compounds are retained, resulting in 1.2 million deaths in 2017, a number that is expected to grow to four million by 2040 [1]. Although transplantation is the best option for ESRD patients, the scarcity of organ donors makes kidney replacement therapies, such as hemodialysis (HD), the next best option [2]. In general, ESRD patients perform HD sessions (ca. 4 h each) three times a week, a regimen that must be followed until a successful transplant is achieved. During an HD session, blood is processed in a hemodialyzer (artificial kidney), an extracorporeal circuit composed of semi-permeable membranes which are responsible for the exclusion of accumulated toxins and excess water while retaining vital blood components such as blood cells, platelets, and proteins. The European Uremic Toxin Work Group (EUTox) is a research team which focuses essentially on identifying solute retention and removal in ESRD patients, and on the deleterious impact of uremic toxins (UTs) on biological systems [3]. Current HD therapy ensures the efficient removal of low molecular weight (<500 Da) water-soluble compounds, such as urea, creatinine, and uric acid. However, other low molecular compounds, known as protein-bound uremic toxins (PBUTs), are responsible for the most severe CKD complications such as cardiorenal syndrome [4] and chronic ischemic heart disease [5]. This is the case of indoxyl sulfate (IS) and *p*-cresyl sulfate (pCS), whose increased levels have been associated with oxidative stress and enhanced expression of inflammatory genes, leading to high hospitalization and mortality rates [6,7,8]. Free PBUTs, which are not bound to proteins, are easily removed by commercial HD membranes by size exclusion, but their high affinity for plasma proteins, especially for human serum albumin (HSA), preclude its removal using the currently available systems. Recent studies have proposed competitive binding as a strategy to remove PBUTs using pharmaceutical drugs as displacers [9,10,11]. Interestingly, in clinical studies ibuprofen (IBF) was found to be the most effective PBUT displacer [12,13]. However, side effects of long-term administration of IBF (and other pharmaceutical drugs) have detrimental effects on the patient’s health, and alternatives to high-dose administration are highly desirable.

Regenerated cellulose has been used as a membrane material since the very early days of dialysis therapy [14]. Despite being strongly hydrophilic, the first generation of hemodialysis membranes exhibited very small apparent pore sizes and were associated with the activation of the complement system rendering hemodialyzers with inefficient mass transfer characteristics and poor hemocompatibility [15]. Because cellulose is a low-cost polymer and one of the most abundant and available bio-renewable materials, researchers were quick to develop modified cellulose materials such as cellulose acetate (CA) and cellulose triacetate where hydroxyl groups are replaced with acetate radicals [16], eliminating the active surface sites for complement protein interaction and, therefore, enhancing hemocompatibility [17]. This modification also rendered membranes with increased average pore size, slightly higher hydraulic permeability (*L_p_*), and a broader solute removal spectrum for CA in comparison to unsubstituted cellulosic membranes [18].

Despite being more efficient than regenerated cellulose, CA membranes present disadvantages such as low thermal and mechanical properties. Low shelf-life and resistance to environmental degradation are also still major drawbacks. To overcome these limitations, Mendes et al. [19] developed cellulose acetate/silica (CA/SiO_2_) monophasic hybrid membranes (MHMs) where the covalent bonding between the inorganic (silica) compounds and the organic (cellulose acetate) matrix is achieved by an innovative method that couples phase inversion and sol-gel techniques. Integral asymmetric MHMs, characterized by a very thin, dense active layer and a much thicker, porous support layer containing up to 40 wt.% of SiO_2,_ were developed using the silicon precursor tetraethyl orthosilicate (TEOS). Permeation tests revealed that the incorporation of SiO_2_ increased the *L_p_* and molecular weight cut-off (MWCO) values, 59 kg/h/m^2^/bar and 111 kDa, respectively, by a factor of two, when compared to the pristine CA membrane (32 kg/h/m^2^/bar and 54 kDa) [19].

Hemocompatibility assays, following the international standard ISO10993-4:2002, performed on the CA/SiO_2_ membranes containing up to 18 wt.% of SiO_2,_ revealed that all the membranes were non-hemolytic and presented lower thrombosis degrees and levels of platelet adhesion and activation when compared to the pristine CA membrane. This finding indicated that the incorporation of SiO_2_ enhances hemocompatibility, making MHMs good candidates for blood-contacting applications [20]. 

In terms of permeation performance, the CA/SiO_2_ membranes were classified as suitable for high-flux hemodialysis, fully permeated urea, creatinine, uric acid (surrogate markers of low molecular weight water-soluble compounds (LMWMs)), and *p*-cresyl sulfate (a surrogate marker of PBUTs) while completely rejecting albumin (a vital component of the blood which cannot be removed by hemodialysis membranes) [21]. Andrade et al. [22] developed another group of MHMs, CA/SiO_2_/SiO_1.5_-(CH_2_)_3_NH_2_, using two silicon precursors: TEOS (up to 5 mol%) and (3-aminopropyl)-triethoxysilane (APTES) (up to 50 mol%). Permeation tests revealed that the incorporation of APTES resulted in integral asymmetric MHMs with *L_p_* values reaching 69 kg/h/m^2^/bar. When studied under dynamic conditions in a lab-scale hemodialysis circuit, the CA/SiO_2_/SiO_1.5_-(CH_2_)_3_NH_2_ membranes fully permeated urea, creatinine, and uric acid (surrogate markers of LMWMs) and completely rejected albumin [23], proving, once again, their potential for hemodialysis.

The possibility to covalently bond two different silicon precursors, TEOS and APTES, to CA and produce integral asymmetric membranes with enhanced hemocompatibility and able to cover a wide range of ultrafiltration profiles opens the door for the development of novel and more efficient hemodialysis membranes. With the urgent need to find new strategies to enhance PBUTs removal, an approach using MHMs produced by novel silicon precursors incorporating competitive binders (such as IBF) is herein proposed, thus opening new trends for the development of smart, toxin-competitive HD membranes.

## 2. Materials and Methods

### 2.1. Materials

Materials included 4-Isobutyl-α-methylphenylacetic acid (ibuprofen, IBF) (99%, racemic mixture), (3-aminopropyl) triethoxysilane (APTES, ≥98%), methyl red (MR, crystalline), *N*,*N*’-dicyclohexylcarbodiimide (DCC, 99%), 1-hydroxypyrrolidine-2,5-dione (NHS), cellulose acetate (CA, average *M_n_* ~30,000 by GPC), and triethylamine that were purchased from Aldrich. Acetonitrile (HPLC grade) and *n*-hexane (HPLC grade) were purchased from Carlos Erba. Tetraethyl orthosilicate (TEOS, 98%) was acquired from Alfa Aesar. Formamide (for analysis ACS) was purchased from PanReac Applichem (Barcelona, Spain), nitric acid (65%) from Chem-Lab (https://www.chem-lab.be/en-gb, accessed on 22 December 2022), and pure acetone from José Manuel Gomes dos Santos, Lda (Odivelas, Portugal). All solvents were used as received. 

### 2.2. Silicon Precursors Synthesis

#### 2.2.1. Conjugation of MR to APTES (MR-APTES)

Following a modified protocol [24], APTES (41.2 mg, 0.186 mmol) was added to MR (50.0 mg, 0.186 mmol) in 5 mL of acetonitrile. DCC (57.6 mg, 0.279 mmol), NHS (32.1 mg, 0.279 mmol), and triethylamine (28.2 mg, 38.8 μL, 0.279 mmol) were added to the mixture and left under magnetic stirring for 8 h at room temperature. After this period, the mixture was filtered to remove unreacted MR, and the residue was washed several times with acetonitrile. The solvent was evaporated to dryness to afford MR-APTES as a red-brown solid. Residual dicyclohexylurea, the conjugation by-product, was observed in the ^1^H NMR spectra. ^1^H NMR (300 MHz, DMSO) δ (ppm): 7.78–7.52 (m, 3H), 7.49–7.22 (m, 3H), 6.83 (d, 2H, *J* = 9.0 Hz), 3.72 (q, 6H, *J* = 6.0, 3.0 Hz), 3.07 (s, 6H), 1.16–1.09 (m, 9H), 0.64–0.52 (m, 2H). ATR-FTIR λ (cm^−1^): 3504, 2940, 1737, 1370, 1220, 1161, 1034, 903.

#### 2.2.2. Conjugation of IBF to APTES (IBF-APTES)

APTES (53.6 mg, 0.242 mmol) was added to IBF (50.0 mg, 0.242 mmol) in 5 mL of acetonitrile. DCC (74.9 mg, 0.363 mmol), NHS (41.8 mg, 0.363 mmol), and triethylamine (36.7 mg, 55.6 μL, 0.363 mmol) were added to the mixture and left under magnetic stirring for 8 h. After several washings with acetonitrile, IBF-APTES was obtained as a white solid (91 mg) in a 91% yield. ATR-FTIR λ (cm^−1^): 3290, 2928, 2854, 2362, 1698, 1653, 1574, 1538, 1449, 1376, 1345, 1279, 1228, 1190, 1143, 1084, 890, 850, 784, 646. Residual dicyclohexylurea, the conjugation by-product, was observed in the ^1^H NMR spectra. ^1^H NMR (300 MHz, CDCl_3_) δ (ppm): 7.16 (d, 2H, *J* = 9.0 Hz), 7.09 (d, 2H, *J* = 6.0 Hz), 4.15–3.36 (m, 6H), 2.44 (d, 2H, *J* = 9.0 Hz), 1.82–1.49 (m, 18H), 1.43 (d, 4H, *J* = 6.0 Hz), 1.15–1.07 (m, 9H), 0.87 (d, 6H, *J* = 6.0 Hz).

#### 2.2.3. Conjugation of IBF to TEOS (IBF-TEOS)

Following a modified protocol, IBF (200.0 mg, 0.97 mmol), TEOS (101.0 mg, 0.48 mmol), triethylamine (49.1 mg, 67.6 μL, 0.48 mmol), and water (1 drop) were added to a glass vial containing acetonitrile (1.6 mL) and left for 1 h at room temperature under stirring. After this period, diethyl ether (3 mL) was added, followed by *n*-hexane (2 mL), and the mixture was stored for 48 h. Then, more *n*-hexane was added to precipitate unreacted IBF. After filtration, the filtrate was evaporated to give IBF-TEOS as a colorless oil (252.2 mg) with an 84% yield. ATR-FTIR λ (cm^−1^): 2956, 2871, 2362, 1723, 1562, 1512, 1459, 1386, 1212, 1174, 1054, 882, 854, 788, 713, 657, 638. ^1^H NMR (300 MHz, CDCl_3_) *δ* (ppm): 7.28 (d, 4H, *J* = 6.0 Hz, superimposed with the CDCl_3_ peak), 7.08 (d, 4H, *J* = 6.0 Hz), 3.91–3.64 (m, 4H), 2.95 (q, 4H, *J* = 18.0, 9.0 Hz), 2.45 (d, 4H, *J* = 6.0 Hz), 1.87–1.81 (m, 2H), 1.49 (d, 6H, *J* = 6.0 Hz), 1.28–1.24 (m, 4H), 1.12 (t, 6H, *J* = 6.0 Hz), 0.91 (d, *J* = 6.0 Hz, 12H). MS ESI (+) (*m*/*z*): 527.20 (100.0%), 528.31 (34%), 530.29 (16%) (M-1).

### 2.3. Membranes Preparation

Five membranes were prepared: a pure cellulose acetate membrane (CA100) and four monophasic hybrid integral asymmetric cellulose acetate/silica membranes containing the synthesized silicon precursors (IBF-ATPES, MR-APTES, and IBF-TEOS).

#### 2.3.1. Pure Cellulose Acetate Membrane (CA100)

The casting solution was prepared by adding CA (4.25 g, 0.142 mmol) to formamide (7.50 g, 166.5 mmol) and acetone (13.25 g, 228.1 mmol) in a glass vial that was sealed, and the mixture was stirred at room temperature for 24 h. Then, following the phase inversion method [25], the casting solution was cast on a glass plate using a 250 µm Gardner casting knife and a solvent evaporation time of 30 s, after which it was quenched in a coagulation bath containing water between 0–5 °C. After approximately 30–60 min., the membranes were detached from the glass plate and stored in deionized water at 4 °C.

#### 2.3.2. Monophasic Hybrid Integral Asymmetric Cellulose Acetate/Silica Membranes (CA/TEOS/APTES)

These membranes were prepared by coupling the phase inversion method with the sol-gel technique [22]. Briefly, the casting solutions were prepared in two steps. First, CA, formamide, and acetone were mixed in a reaction vessel to allow the complete dissolution of CA. Following complete solubilization of CA, TEOS, and/or APTES, and the silica precursor (IBF-TEOS, IBF-APTES or MR-APTES), three drops of nitric acid were added to the mixture, promoting hydrolysis and hetero-condensation during the casting solution homogenization step. The final solutions were placed on an agitation plate for 24 h. After the phase inversion step described above for CA100, the membranes were stored in deionized water at 4 °C. The composition of each membrane is shown in Table 1. 

### 2.4. Precursors and Membranes Characterization

The NMR spectra were recorded on Bruker ARX 400 MHz equipment. The ^1^H chemical shifts are reported as ppm (parts per million). The UV–Vis spectra were obtained in a UV-1700 PharmaSpec Spectrometer from Shimadzu (Canby, OR, USA) over the spectral range of 200 to 900 nm with a scan rate of 100 nm min^−1^ at 25 °C in 1.0 cm cuvettes. Attenuated Total Reflectance-Fourier Transform Infrared (ATR–FTIR) spectroscopy was used to analyze the active layer of each membrane. The equipment used was a Nicolet 5700 FT-IR spectrometer, from Thermo Electron Scientific Instruments (Madison, WI, USA), with a Golden Gate MKII ATR accessory with a Ge crystal (Graseby Specac, Smyrna, GA, USA; sampling depth: 0.2–1.1 µm at 4000–600 cm^−1^). The ATR-FTIR spectra were obtained from one sample of each composition by averaging 264 scans with a resolution of 4 cm^−1^ and processing using the OMNIC™ software from Thermo Fisher Scientific. Mechanical agitation in membrane synthesis was improved by the Shaker S50 purchased from CAT (Irving, TX, USA). Samples of the dry membranes were broken in liquid nitrogen, mounted on a stub, and gold-sputtered prior to being analysed by Scanning Electron Microscopy (SEM) using the equipment Thermo Scientific™ Phenom™ ProX G6 desktop SEM. The average thickness and respective standard deviations were measured from six different regions of the cross-section, using the software ImageJ version 2.3.0 developed by NIH.

### 2.5. Leaching Assay

To evaluate the possible leaching of the competitive binder added via the silicon precursor, we evaluated the leaching of MR (model compound) from the membrane. A sheet of CA/TEOS/APTES-MR membrane was stored in deionized water, samples of the storage water were periodically collected for a month, and the UV-Vis absorption was recorded at 410 nm (MR λ_max_).

## 3. Results and Discussion

### 3.1. Silicon Precursors Synthesis

**Scheme 1:**
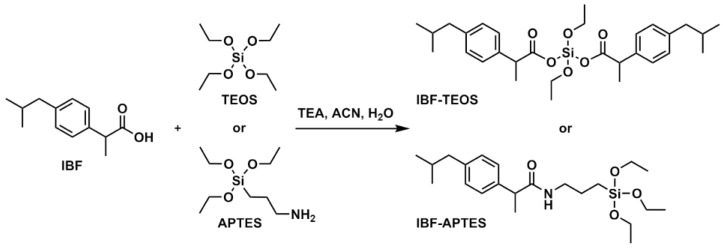
Conjugation of ibuprofen (IBF) with (3-aminopropyl) triethoxysilane (APTES) and tetraethyl orthosilicate (TEOS). TEA = triethylamine; ACN = acetonitrile.

The membranes developed in this study were designed to strongly bind HSA. Therefore, the drug-conjugated membrane triggers a competitive binding, a stimulus that enables the smart displacement of PBUTs that usually bind to HSA. Based on previous studies that demonstrated the high binding affinity of IBF towards HSA [12,13], in this work, IBF was conjugated both to TEOS and APTES following reactions represented in Scheme 1. This would allow IBF incorporation into monophasic hybrid asymmetric cellulose acetate/silica membranes.

**Scheme 2:**
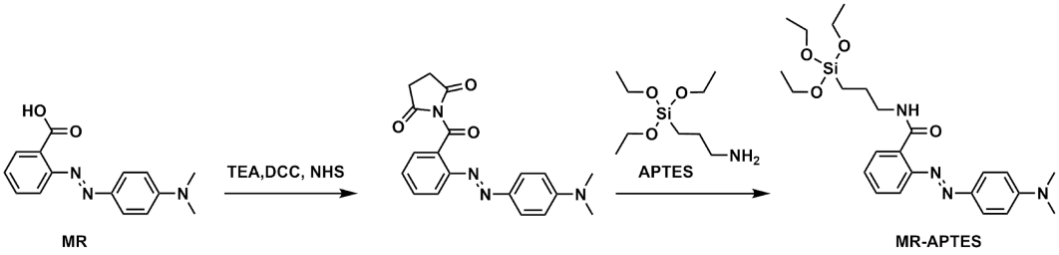
Conjugation of methyl red (MR) with (3-aminopropyl) triethoxysilane (APTES). TEA = triethylamine; DCC = *N*,*N*’dicyclohexylcarbodiimide; NHS = 1-hydroxypyrrolidine-2,5-dione.

In the conjugation of IBF with TEOS only the disubstituted product was obtained (Scheme 1) [26], a result confirmed by ^1^H NMR (Figure S1) and mass spectrometry (Figure S2). In the conjugation of IBF with APTES, only the monosubstituted product was obtained (Figure S3). As a proof of concept, MR, which like IBF has a carboxylic acid functional group, was selected as a model compound in our studies. An organic dye, MR allows a ready visualization of its incorporation into the membrane. Following a modified reported protocol [24], MR was conjugated to APTES (Scheme 2). 

The ^1^H NMR spectrum also confirmed the successful conjugation of MR to APTES (Figure S4). The structure of the silicon precursors was confirmed by ATR-FTIR, where the bands observed at 1737 (MR-APTES), 1698 (IBF-APTES), and 1723 (IBF-TEOS) cm^−1^ clearly identified the presence of the corresponding carbonyls. 

### 3.2. Membranes Synthesis

Three monophasic hybrid integral asymmetric cellulose acetate/silica membranes using the synthesized silicon precursors were produced: CA/TEOS/MR-APTES-2 (2%), CA/TEOS/IBF-APTES-3 (3%), CA/IBF-TEOS/APTES-9 (9%), and CA/IBF-APTES-15 (15%). The indicated percentage is the mass percentage relative to the total mass of CA, TEOS, APTES, and the silicon precursor (excluding formamide and acetone). 

**Scheme 3:**
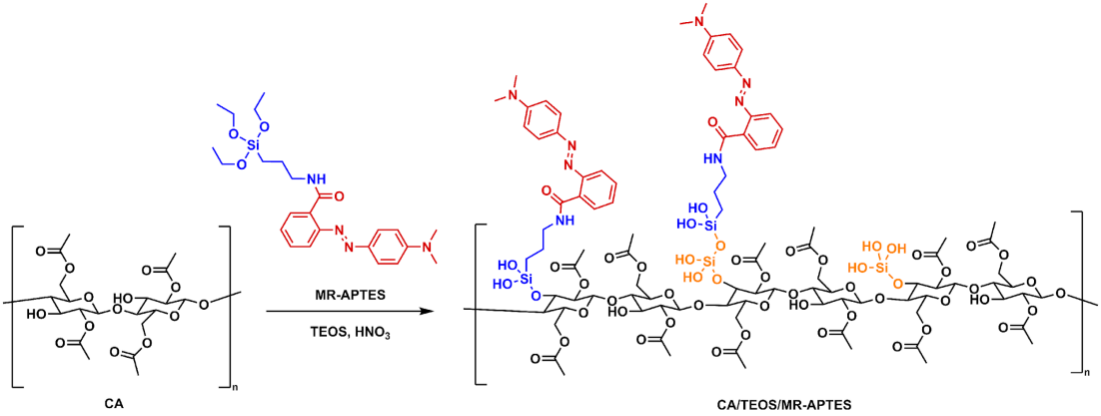
Schematic representation of the sol-gel reaction between cellulose acetate (CA), MR-APTES, and TEOS to obtain CA/TEOS/MR-APTES membranes. Color scheme: TEOS in orange, MR in red, and APTES in blue.

Following the well-established protocol by Faria et al. [19,21] for the preparation of the ultrafiltration membranes where through a sol-gel reaction the silicon derivatives TEOS and APTES can be covalently bonded to cellulose acetate, a membrane containing 2% (wt) of MR-APTES was prepared (Scheme 3). 

**Figure 1:**
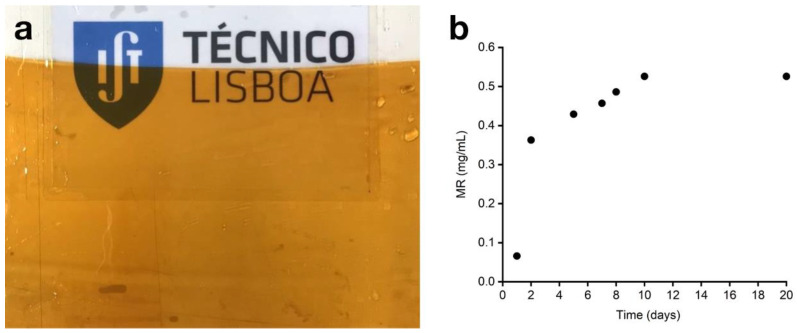
Monophasic hybrid integral asymmetric CA/TEOS/MR-APTES-2 membrane: Picture showing the color and homogenous distribution of the silicon precursor (MR-APTES) (**a**), and cumulative release of methyl red (MR) to the storage water (**b**).

Regarding the CA/TEOS/MR-APTES-2 membrane, with only 2% of MR, it was possible to visually observe the homogeneous distribution of the silicon precursor through the membrane, without the formation of visible aggregates (Figure 1a). Moreover, leaching studies performed for 20 days showed a residual release of MR into the storage water (0.66% of cumulative release) (Figure 1b).

**Figure 2:**
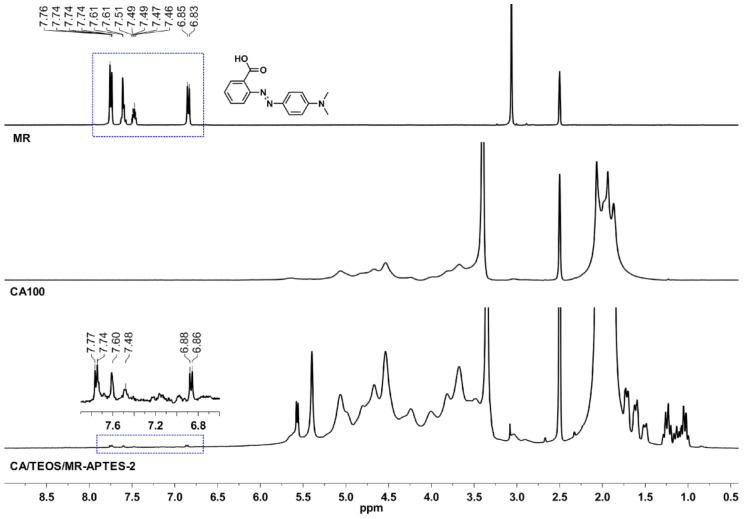
^1^H NMR spectra of methyl red (MR), CA100 membrane, and CA/TEOS/MR-APTES-2 membrane in DMSO-*d*6.

Moreover, the stability and the integrity of the CA/TEOS/MR-APTES-2 membranes were evaluated for approximately one year and were found to keep their properties, without MR being detected in the storage water. Since these membranes are highly soluble in dimethyl sulfoxide (DMSO), their stability was also evaluated by ^1^H NMR. Contrary to MR, CA does not possess aromatic protons. Therefore, by comparing the spectra obtained for the CA100 and the CA/TEOS/MR-APTES-2 membranes, it is very clear that the peaks between 7.77–6.86 ppm correspond to the aromatic ring of MR, giving evidence that the silicon precursor is incorporated in the membrane, even almost one year after being prepared (Figure 2).

**Scheme 4:**
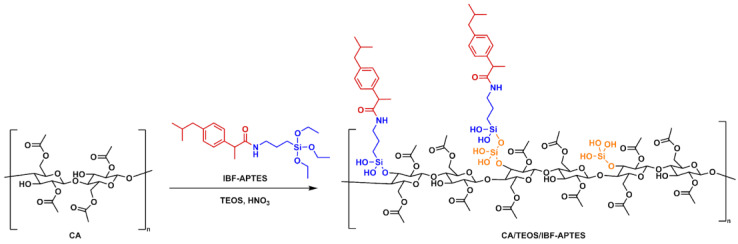
Schematic representation of the sol-gel reaction between cellulose acetate (CA), IBF-APTES, and TEOS to obtain CA/TEOS/IBF-APTES membranes. Color scheme: TEOS in orange, IBF in red, and APTES in blue.

**Scheme 5:**
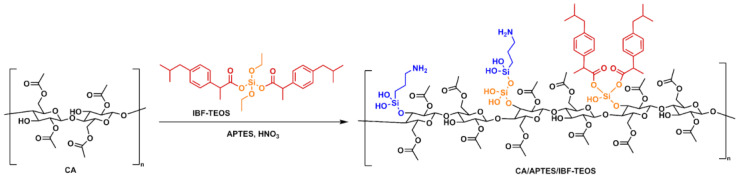
Schematic representation of the sol-gel reaction between the cellulose acetate (CA), IBF-TEOS, and APTES to obtain the CA/APTES/IBF-TEOS membrane. Color scheme: TEOS in orange, IBF in red, and APTES in blue.

Similarly, the CA/TEOS/IBF-APTES-3 and CA/-IBF-TEOS/APTES-9 membranes containing 3% and 9% wt of IBF, respectively, were fabricated by the sol-gel reaction where both silica precursors were mixed with cellulose acetate under acidic conditions (Scheme 4 and Scheme 5). 

**Figure 3:**
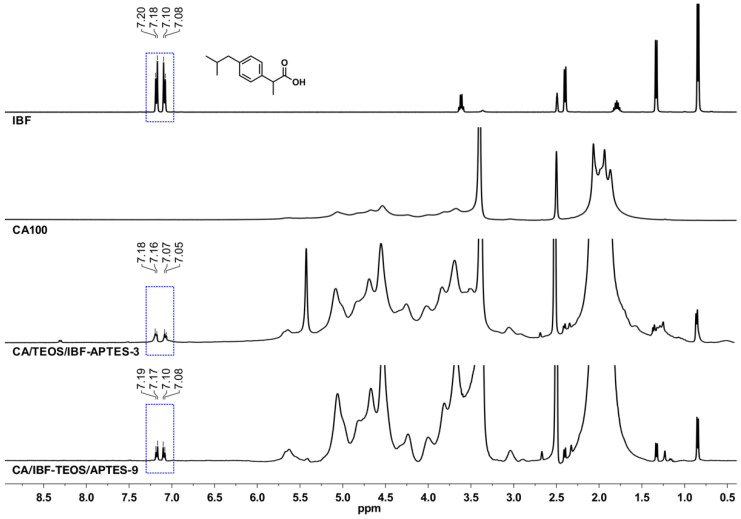
Comparison of the ^1^H NMR spectra of ibuprofen (IBF), CA100 membrane, CA/TEOS/IBF-APTES-3 membrane, and CA/IBF-TEOS/APTES-9 membrane in DMSO-*d*6.

The stability and integrity of the CA/TEOS/IBF-APTES-3 and CA/-IBF-TEOS/APTES-9 membranes were also evaluated by ^1^H NMR after several months of being produced. Figure 3 clearly demonstrates that in both membranes the aromatic protons of IBF are present in the region 7.19–7.08 ppm, thus confirming its incorporation in the membranes.

**Figure 4:**
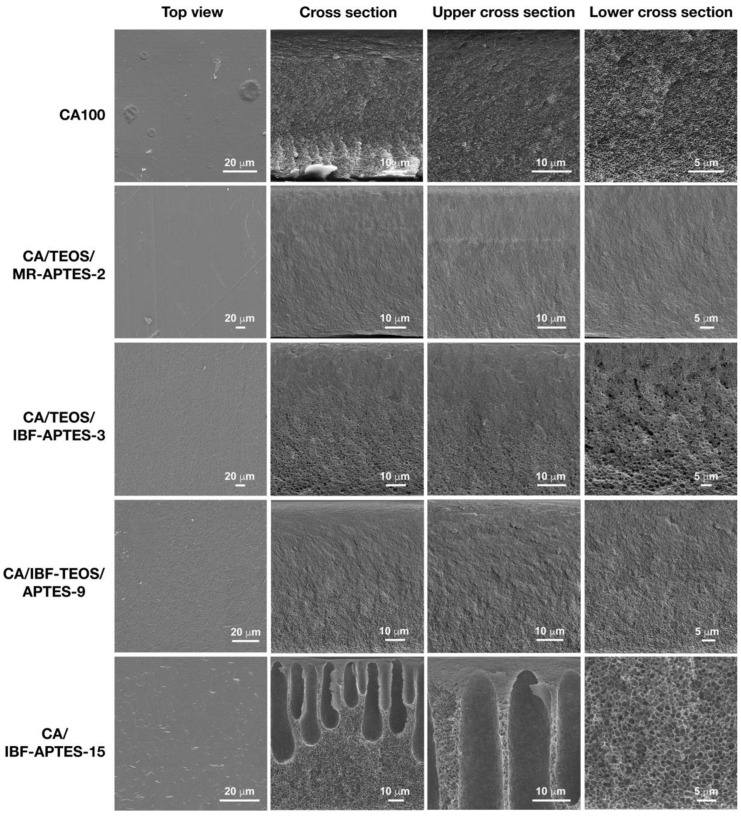
SEM images of the produced membranes.

Figure 4 shows the SEM micrographs obtained for the dense active layer surface and the cross-section of the dry membranes. In each case, it is possible to observe a top-dense active layer. The cross-section images confirmed the presence of a very thin skin layer and, beneath it, a thicker, porous substructure in the lower part of the membrane. This microstructure is due to the efficiency of the phase inversion method used in the fabrication of these membranes. The SEM images of the CA/IBF-APTES-15 membrane show the formation of finger-like macropores. However, a higher percentage of silicon precursor (15%) in the membrane composition does not affect the membrane’s skinned asymmetric character.

Table 2 summarizes the overall thickness of each membrane, estimated from the cross-section images. According to previous studies [20,22,23], the introduction of silicon precursors, such as TEOS and APTES, resulted in the formation of thicker membranes compared to the pure CA membrane. In this work, this tendency was also observed, since thicker membranes were obtained when the silica content was increased.

## 4. Conclusions

The urgent need to find new strategies to enhance the removal of PBUTs has drawn the attention of membrane scientists in the past few years. In this work, we present a novel approach that combines the properties of integral asymmetric monophasic hybrid cellulose acetate/silica membranes and the power of IBF as a strong HSA binding competitor towards producing HD membranes which target the removal of PBUTs and prevent the infusion of high IBF dosage to patients under HD treatment. Two novel silicon precursors containing IBF, IBF-TEOS, and IBF-APTES were synthesized and reacted with CA to produce three different IBF-conjugated smart membranes (HSA responsive), showing high stability over large periods of storage. By ^1^H NMR analysis, it was possible to observe the presence of the silicon precursors in the membranes, even in low percentages. The SEM analysis also demonstrates that it is possible to maintain the integrity of the membrane, even for a membrane with a higher silicon percentage (e.g., CA/IBF-APTES-15 membrane).

We also used the methyl red (MR) dye as an IBF surrogate to evaluate the incorporation of IBF in the membranes, using a simple and straightforward colorimetric strategy. The versatility of our approach enables the incorporation of other target drugs, thus enabling the production of tailored smart membranes with competitive binding properties for highly efficient future hemodialyzers, thus avoiding supplementary drug administration.

## Figures and Tables

**Table 1:** Composition of the monophasic hybrid integral asymmetric cellulose acetate/silica membranes.

	Membrane Composition (g)			
	CA/TEOS/ MR-APTES	CA/ IBF-TEOS/ APTES	CA/TEOS/ IBF-APTES	CA/ IBF-APTES
Cellulose acetate	4.10	4.10	3.95	15.87
Formamide	7.25	7.25	6.98	20.54
Acetone	12.78	12.78	12.30	56.85
TEOS	0.60	–	1.20	–
APTES	–	0.16	–	–
Nitric acid	0.31 (3 drops)	0.31 (3 drops)	0.31 (3 drops)	0.31 (3 drops)
Silicon precursor	0.16	0.60	0.32	6.74

**Table 2:** Average thickness and standard deviation of each membrane, obtained from the cross-sections in the SEM images.

Membrane	Thickness, ℓ, µm
CA100	52.0 ± 1.2
CA/TEOS/MR-APTES-2	72.5 ± 1.1
CA/TEOS/IBF-APTES-3	78.6 ± 0.6
CA/IBF-TEOS/APTES-9	72.8 ± 0.8
CA/IBF-APTES-15	95.4 ± 1.6

## Data Availability

Not applicable.

## Supplementary Materials

The following supporting information can be downloaded at: https://www.mdpi.com/article/10.3390/jfb14030138/s1, Figure S1: 1H NMR spectra of TEOS, IBF, and the silicon precursor IBF-TEOS in CDCl3; Figure S2: MS spectra of IBF-TEOS; Figure S3: 1H NMR spectra of APTES, MR, and the silicon precursor MR-APTES in DMSO-*d*6; Figure S4: 1H NMR spectra of APTES, IBF, and the silicon precursor IBF-APTES in DMSO-*d*6.
