# Supplementary material for: A Novel Strategy for Enhanced Sequestration of Protein-Bound Uremic Toxins Using Smart Hybrid Membranes
Source: J Funct Biomater. 2023 Feb 28;14(3):138. doi: 10.3390/jfb14030138 (PMC10059720; doi:10.3390/jfb14030138)
Supplement: Supplementary file 1 [file jfb-14-00138-s001.zip › jfb-2150168-Supplementary Materials.pdf]

Supplementary Materials:

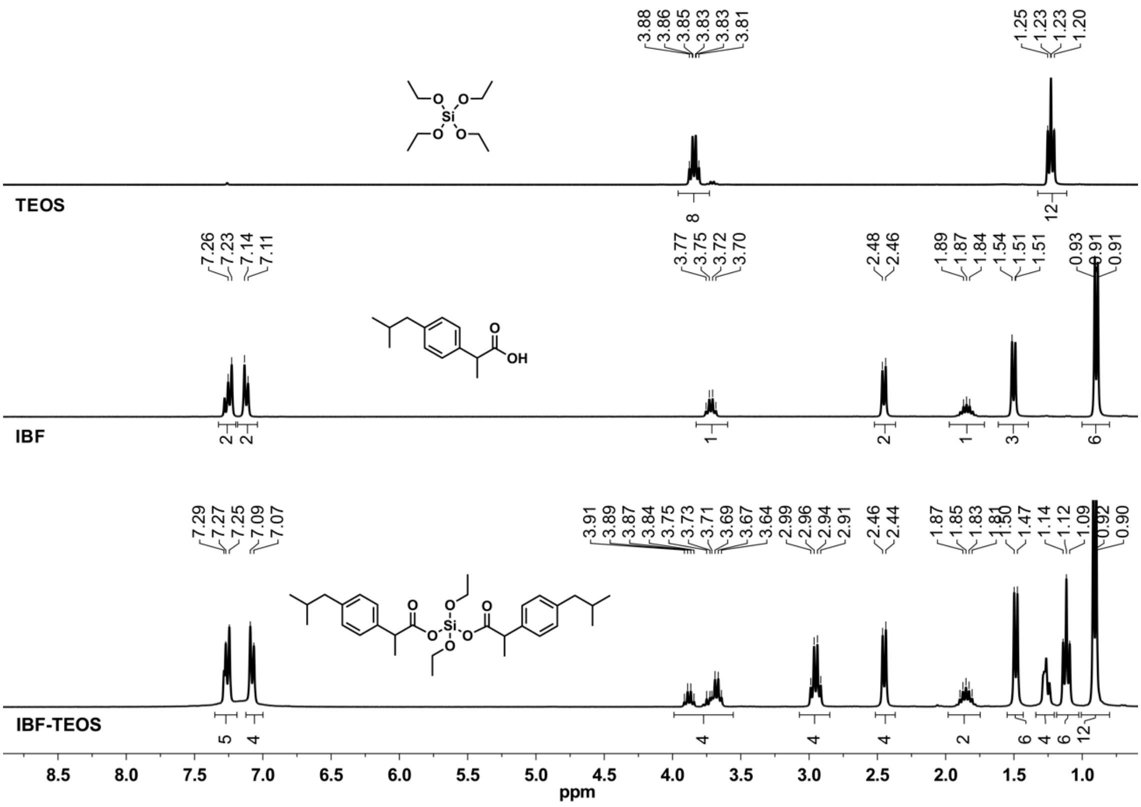

Figure S1. ^{1}H NMR spectra of tetraethyl orthosilicate (TEOS), ibuprofen (IBF) and the silicon precursor IBF-TEOS in $CDCl_3$.

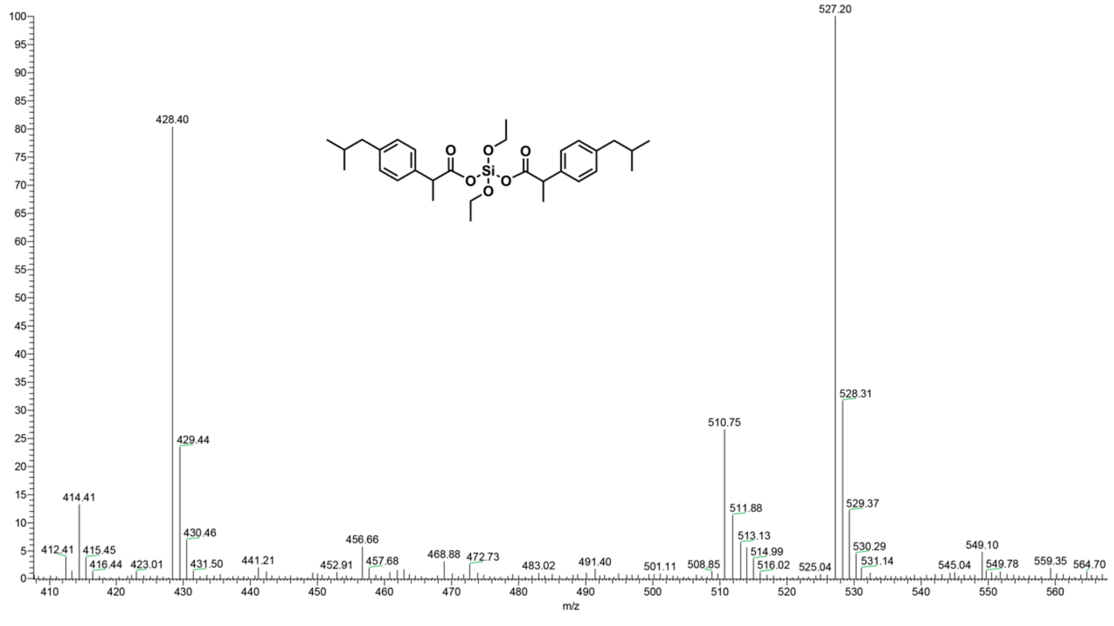

Figure S2. ESI-MS spectrum of IBF-TEOS.

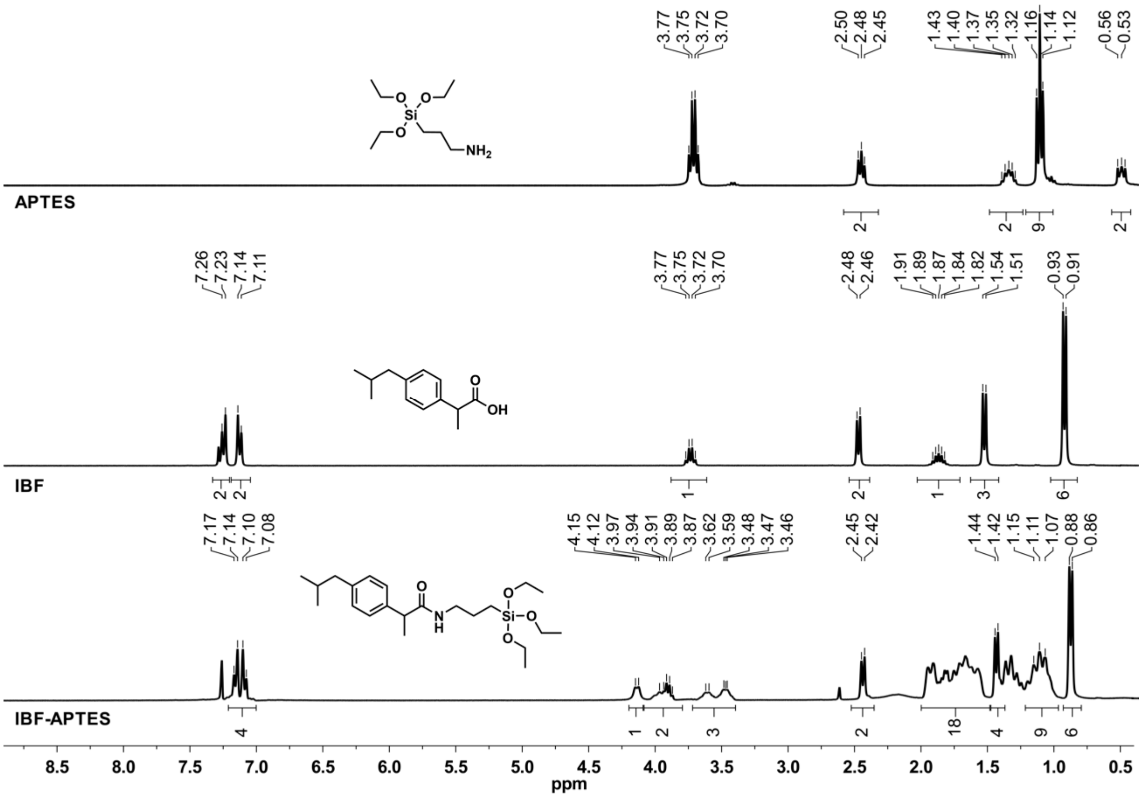

Figure S3. ^{1}H NMR spectra of (3-aminopropyl) triethoxysilane (APTES), ibuprofen (IBF) and the silicon precursor IBF-APTES in DMSO-*d6*.

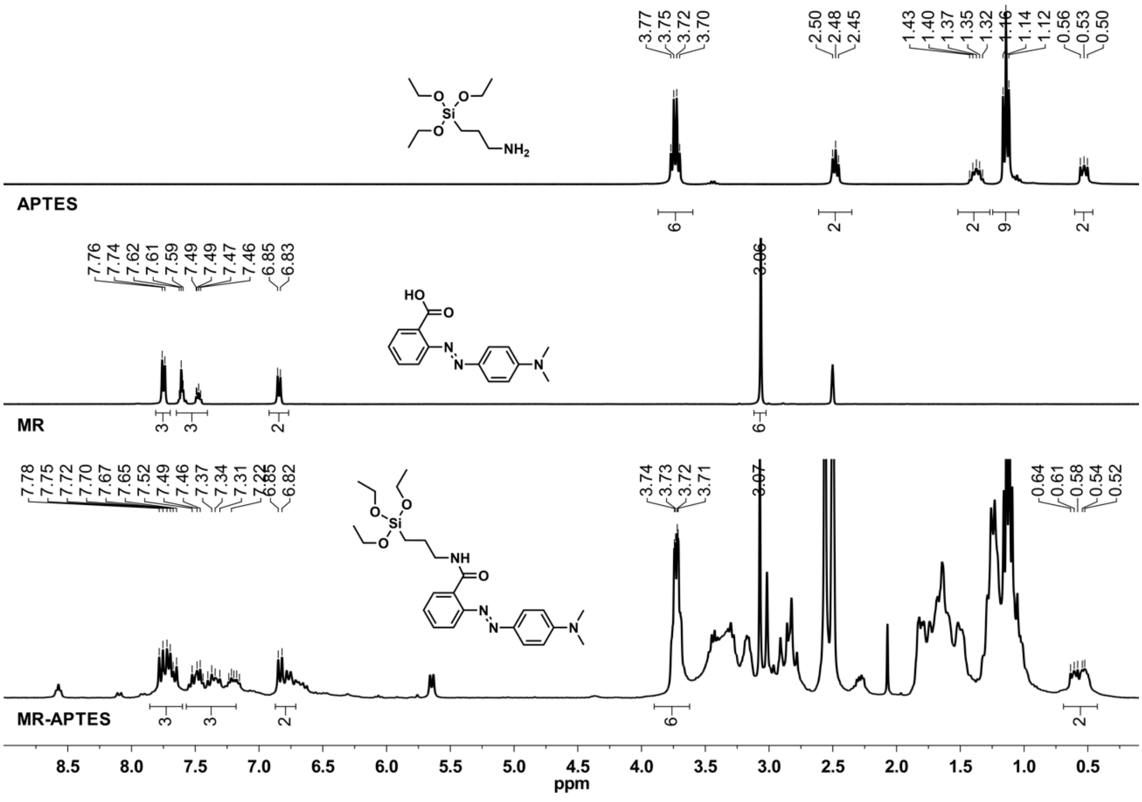

Figure S4. ^{1}H NMR spectra of (3-aminopropyl) triethoxysilane (APTES), methyl red (MR) and the silicon precursor MR-APTES in DMSO-*d6*.
